# Comparing four training modalities on body composition, cardiorespiratory fitness and cardiovascular responses in sedentary young men: a randomized trial

**DOI:** 10.1186/s13102-026-02027-7

**Published:** 2026-08-27

**Authors:** Weihua Zheng, Shuai Han, Shunzhe Piao, Runrong Tang, Hansen Li, Weipeng Liu, Yang Cao

**Affiliations:** 1 School of Physical Education, Jimei University, Xiamen, China; 2 School of Social Sports, Shenyang Sport University, Shengyang, China; 3 No.113 Middle School of Guangzhou, Guangzhou, China; 4 Yunshan School of Huadu District, Guangzhou, China; 5 College of Physical Education, Sichuan Agricultural University, Yaan, 625014 China; 6 School of Strength and Conditioning, Shenyang Sport University, Shenyang, China; 7 Clinical Epidemiology and Biostatistics, School of Medical Sciences, Faculty of Medicine and Health, Örebro University, Örebro, 70182 Sweden; 8 Unit of Integrative Epidemiology, Institute of Environmental Medicine, Karolinska Institutet, Stockholm, 17177 Sweden

**Keywords:** High-intensity interval training, High-intensity continuous training, Moderate-intensity interval training, Moderate-intensity continuous training, Sedentary young men

## Abstract

**Background:**

Although high-intensity interval training (HIIT), high-intensity continuous training (HICT), moderate-intensity interval training (MIIT), and moderate-intensity continuous training (MICT) have all been shown to improve health outcomes, few studies have compared these four modalities within the same framework. Therefore, this study aimed to compare the effects of 8 weeks of HIIT, HICT, MIIT, and MICT on body composition, cardiorespiratory fitness, and cardiovascular responses in sedentary young men. We hypothesized that high-intensity training would produce greater improvements than moderate-intensity training.

**Methods:**

Forty sedentary men aged 20-28 years were randomized to the HIIT, HICT, MIIT, or MICT group, of whom 38 completed the intervention, corresponding to an attrition rate of 5%. Participants completed three approximately 45-minute training sessions per week for 8 weeks, with exercise intensity prescribed at 77%-95% of maximal heart rate (HRmax) and a rating of perceived exertion (RPE) of 14-17 for the HICT and HIIT groups and at 64%-76% HRmax and an RPE of 12-13 for the MICT and MIIT groups, while exercise duration was matched across groups at 120 seconds per movement. The primary outcomes were VO2peak and cardiovascular responses, including resting and peak-exercise heart rate, systolic and diastolic blood pressure, and rate-pressure product. Secondary outcomes included total exercise duration to volitional exhaustion during the Bruce graded exercise test (Bruce test duration) and body-composition measures. Group-by-time interactions were examined using linear mixed-effects models, followed, where appropriate, by Bonferroni-adjusted post hoc comparisons.

**Results:**

Significant group-by-time interactions were observed for VO2peak (P = 0.040, η2 = 0.21), Bruce test duration (P = 0.016, η2 = 0.26), resting systolic blood pressure (P < 0.001, η2 = 0.44), resting rate–pressure product (P < 0.001, η2 = 0.40), peak-exercise systolic blood pressure (P = 0.001, η2 = 0.36), and peak-exercise rate–pressure product (P < 0.001, η2 = 0.42). In contrast, the interactions were nonsignificant for resting heart rate (P = 0.064, η2 = 0.21), resting diastolic blood pressure (P = 0.615, η2 = 0.05), peak-exercise heart rate (P = 0.078, η2 = 0.18), and peak-exercise diastolic blood pressure (P = 0.220, η2 = 0.12). Bonferroni-adjusted comparisons showed a greater increase in VO2peak with HIIT than with MIIT (MD = 1.11 mL·kg-1·min-1; 95% CI [0.20, 2.01], g = 1.54; P < 0.01), whereas no adjusted between-group differences were detected for Bruce test duration. The largest differences in cardiovascular responses were observed between HICT and MIIT for resting systolic blood pressure (MD= -4.95 mmHg; 95% CI [-7.65, -2.25], g = -1.70), resting rate–pressure product (MD= -0.80 mmHg·bpm; 95% CI [-1.18, -0.42], g = -1.61), and peak-exercise systolic blood pressure (MD= -6.07 mmHg; 95% CI [-10.38, -1.76], g = -1.94), whereas the largest difference in peak-exercise rate–pressure product was observed between HIIT and MIIT (MD= -0.77 mmHg·bpm, 95% CI [-1.26, -0.28], g = -1.79). No group-by-time interactions were detected for body weight (P = 0.985), fat mass (P = 0.950), fat-free mass (P = 0.977), body mass index (P = 0.924), or visceral adipose tissue (P = 0.273).

**Conclusions:**

These preliminary findings suggest outcome-specific differences among the training protocols. HIIT improved VO2peak more than MIIT, while high-intensity protocols showed greater improvements in selected cardiovascular responses. No adjusted between-group differences were detected for Bruce test duration, and no differential effects were observed for body composition. Larger controlled trials are needed to confirm these findings.

**Trial registration:**

Chinese Clinical Trial Registry ChiCTR2500106395. Registered 23 July 2025.

**Supplementary Information:**

The online version contains supplementary material available at https://doi.org/10.1186/s13102-026-02027-7.

## Introduction

Sedentary behavior is defined as any waking activity with an energy expenditure of ≤ 1.5 metabolic equivalents [1]. Currently, sedentary behavior has become a global public health challenge. Studies have confirmed that prolonged sedentary behavior significantly increases the risk of obesity, various metabolic and cardiovascular diseases, and even cancer [1–3]. This phenomenon is increasingly affecting younger populations, and excessive obesity can markedly increase the burden on the cardiovascular and metabolic systems. This may lead to a decline in cardiorespiratory endurance in young individuals and is closely associated with hypertension [4–7]. At the same time, given that there is a positive relationship between body composition and the cardiovascular system, a favorable body composition contributes to better cardiovascular function. In addition, the maintenance of cardiovascular health is a key factor in promoting healthy lifespan and longevity [8, 9]. Therefore, it is essential to improve both body composition and cardiorespiratory fitness. Although the World Health Organization (WHO) recommends that adults engage in at least 75 min of high intensity exercise or 150 min of moderate intensity aerobic exercise per week [10], many individuals find it difficult to meet these guidelines due to time constraints, limitations in ongoing health conditions, and restricted access to exercise facilities [11–13]. Therefore, the field of exercise science has been attempting to propose exercise prescriptions from multiple perspectives to help the general population break sedentary behavior.

Moderate-intensity continuous training (MICT), which is widely recognized, typically refers to continuous aerobic exercise performed at approximately 46% to 63% of maximal oxygen uptake (VO_2_max) or 64% to 76% of maximal heart rate (HR_max_). Its effects on improving body composition and cardiorespiratory fitness have been extensively supported by previous studies [14–17], However, its relatively low adherence remains a major limitation in clinical practice [18]. When young individuals withdraw from training programs due to time constraints or lack of interest, the effectiveness of exercise prescriptions is substantially reduced [19, 20], This has prompted the exploration of more time-efficient and enjoyable intervention modalities.

In this context, high intensity interval training (HIIT) has gradually gained increasing attention. HIIT typically refers to an intermittent exercise modality performed at an intensity of approximately 64% to 90% VO_2_max or 77% to 95% HR_max_. It involves alternating periods of short-duration high-intensity effort and recovery, and is characterized by high time efficiency and relatively low requirements for facilities and equipment [21]. Previous studies have demonstrated that HIIT has significant benefits in improving body composition, cardiorespiratory endurance, and metabolic function [22]. However, inconsistent findings remain regarding comparisons between HIIT and MICT. Some studies suggest that there are no significant differences between HIIT and MICT in body composition and cardiorespiratory outcomes [23–26], whereas other studies report opposite results [27–29]. These inconsistencies may be related to differences in participant characteristics, intervention duration, training protocols, and approaches to matching training exposure. More importantly, conventional comparisons between HIIT and MICT simultaneously alter both exercise intensity and training format, making it difficult to determine whether observed adaptations are attributable to the higher intensity, the intermittent structure, or a combination of these factors.

Exercise intensity and training format may influence adaptation through related but conceptually distinct pathways. Exercise intensity determines the magnitude of cardiovascular and metabolic stress and may therefore influence oxygen delivery, mitochondrial adaptation, and vascular regulation, whereas interval and continuous formats determine how this physiological stress is accumulated and tolerated during each session. Recovery periods during interval training may facilitate repeated exposure to high-intensity workloads, whereas continuous training may provide a more sustained cardiovascular and oxidative stimulus. Consequently, exercise intensity and training format may exert independent or interactive effects on training adaptation [30–33].

It is worth noting that, in addition to HIIT and MICT, other training modalities, such as moderate-intensity interval training (MIIT) and high-intensity continuous training (HICT), have received relatively limited attention in the existing literature. MIIT can be broadly described as repeated bouts of moderate-intensity exercise, typically corresponding to approximately 64%-76% HRmax, separated by planned recovery periods. In contrast, HICT can be broadly described as sustained high-intensity exercise, typically above approximately 77% HRmax, without systematically prescribed recovery intervals [34]. Evidence regarding MIIT is largely limited to small-sample interventions involving mainly adolescents or adults with overweight or obesity, and inconsistent findings have been reported for body composition, cardiorespiratory fitness, and metabolic outcomes when MIIT is compared with HIIT [35–38]. HICT has been associated with potential benefits for cardiovascular risk factors, cardiorespiratory fitness, and cognitive performance in both healthy and clinical populations [39–43]. Importantly, comparing HICT with HIIT may help determine whether adaptations commonly attributed to HIIT arise primarily from high exercise intensity or from its intermittent work–recovery structure; comparable responses to HICT and HIIT would suggest a greater contribution of exercise intensity, whereas superior responses to HIIT would indicate a potential additional benefit of the interval format. Nevertheless, direct evidence comparing these modalities remains limited and requires further investigation.

At present, HIIT, HICT, MIIT, and MICT have all been associated with improvements in several health outcomes. However, most previous studies have compared only two of these training modalities, and few have examined all four intensity-by-format combinations within the same experimental framework. Furthermore, HIIT and MICT protocols in previous studies have frequently differed not only in exercise intensity and format but also in session duration, total work, or energy expenditure, which may obscure the independent contribution of each factor. Therefore, the present study used a time-matched design in which exercise duration was standardized across groups to reduce potential confounding arising from unequal training exposure, although this strategy did not necessarily equate mechanical work or energy expenditure. The four training conditions represented a conceptual 2 × 2 framework comprising intensity (high versus moderate) and format (interval versus continuous), thereby allowing the potential independent and interactive influences of these factors to be examined.

Accordingly, the present study aimed to compare the effects of 8 weeks of HIIT, HICT, MIIT, and MICT on body composition, cardiorespiratory fitness, and selected cardiovascular responses at rest and during peak exercise in sedentary young men. VO_2_peak was designated as the primary measure of cardiorespiratory fitness. The other primary outcomes comprised selected cardiovascular responses, including resting and peak-exercise heart rate, systolic and diastolic blood pressure, and rate-pressure product. Secondary outcomes included total exercise duration to volitional exhaustion during the Bruce graded exercise test (hereafter, Bruce test duration) and body-composition measures. We hypothesized that high-intensity training would produce greater improvements in cardiorespiratory fitness and selected cardiovascular responses than moderate-intensity training, whereas differences between interval and continuous formats would be smaller. Furthermore, considering that substantial fluctuations in sex hormones during the menstrual cycle in females may potentially interfere with metabolic rate, thermoregulation, and cardiovascular stress responses, and in order to minimize confounding variables and ensure the homogeneity of physiological measurements, only male participants were included in the present study.

## Materials and methods

### Participants

This study was a single center, parallel-group, four arm randomized controlled trial with an 8 weeks intervention. The four intervention groups represented combinations of two training dimensions, namely intensity (high versus moderate) and format (interval versus near-continuous), resulting in HIIT, HICT, MIIT, and MICT conditions.The study protocol strictly followed the ethical principles of the Declaration of Helsinki and the CONSORT reporting guidelines, and was approved by the Ethics Committee of Shenyang Sport University (Approval No. [2025]8) and registered in the Chinese Clinical Trial Registry (ChiCTR2500106395). All participants were fully informed of the study risks and provided written informed consent prior to the experiment. A priori power analysis was conducted using G*Power 3.1 software. A repeated-measures analysis of variance (ANOVA) was specified with an expected effect size of f = 0.3 for within-factor, between-factor, and interaction effects. The significance level was set at α = 0.05, and the statistical power was set at 1 - β = 0.80 [44]. The results indicated that a minimum sample size of 36 participants was required to detect statistically significant effects. Considering potential attrition during the exercise intervention, a larger recruitment target was ultimately determined. The research team conducted broad recruitment among male non-sports major students at a university in Shenyang through electronic campus posters, printed flyers, and social media platforms. Given the high time cost and physical demand of the intervention, a tiered cash incentive system was established to ensure participant adherence. The inclusion criteria were as follows:1) males aged 18 to 28 years; 2) typical sedentary behavior, defined as a total daily sedentary time of ≥ 7 h, and not engaging in regular exercise as confirmed by the International Physical Activity Questionnaire (IPAQ), with less than 150 min of moderate to vigorous physical activity per week [45]; 3) no medical contraindications to exercise, visual impairment, or psychiatric disorders as determined by medical screening; 4) willingness to participate and provision of written informed consent. The exclusion criteria were as follows: (1) participation in other exercise training programs; (2) positive risk indicators identified by the 2020 Physical Activity Readiness Questionnaire for Everyone (PAR-Q+) indicating cardiovascular risk or musculoskeletal pain in the knees or lower back [46]; (3) attendance rate lower than two-thirds of the total intervention sessions.

### Experimental design

**Fig. 1:**
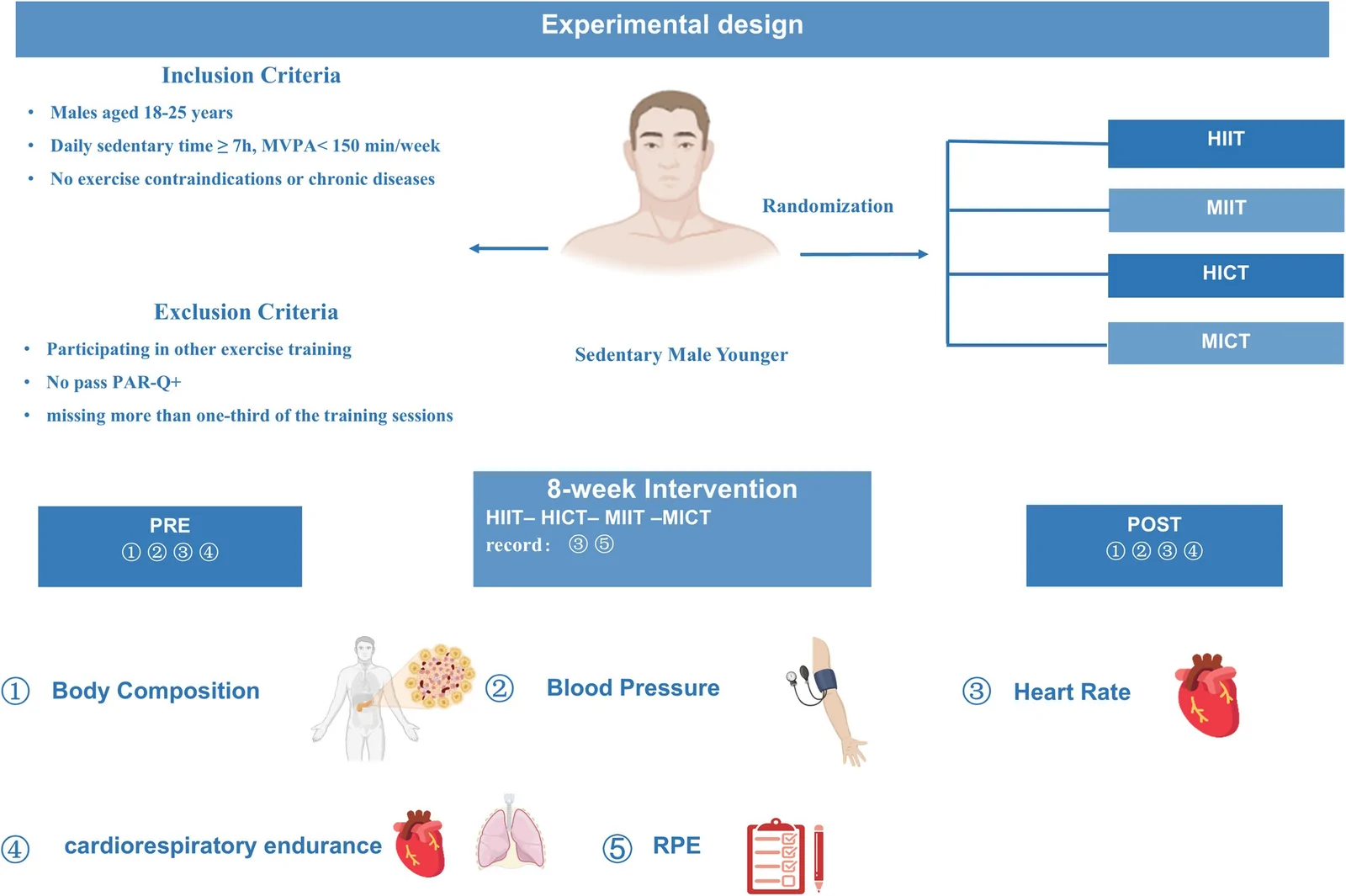
Experimental design process

Eligible participants were randomly assigned to the HIIT group, HICT group, MICT group, and MIIT group using a 1:1:1:1 allocation ratio. To ensure balanced sample sizes across groups, a restricted randomization procedure was adopted. A third-party assistant prepared identical allocation slips indicating group assignments in equal numbers, which were then sealed in opaque envelopes. Furthermore, although participants and training instructors could not be blinded because of the nature of the exercise interventions, data collectors and outcome assessors were blinded to participants’ group assignments during the baseline and post-intervention assessments. After randomization, participants underwent familiarization sessions led by professional assessors to minimize learning effects. Before the intervention, a comprehensive baseline assessment was conducted, including body composition measures (body weight, BMI, fat mass, and others), cardiorespiratory fitness (VO_2_peak, etc.), and cardiovascular function (heart rate, blood pressure, and rate pressure product at rest and during exercise). Subsequently, all variables were reassessed within one week after the completion of the 8 weeks intervention. In addition, to strictly control confounding factors, participants were required to abstain from smoking, alcohol, and caffeine for 48 h before each testing session and to avoid any vigorous physical activity, thereby ensuring that physiological indicators reflected a true baseline state. Finally, the visual flowchart of the study design is presented in Fig. 1.

### Training protocol

Before the formal intervention, all participants completed 2 weeks familiarization period consisting of four sessions, which aimed to help them become familiar with the training movements and master correct techniques. Notably, the initially designed training protocol was evaluated as having excessive intensity during the familiarization phase. Therefore, to ensure the safety and feasibility of the formal intervention, the researchers adjusted the exercise content and workload based on participant feedback and heart rate monitoring data, and the final training protocol is presented in Supplementary S1. After familiarization, participants completed an 8 weeks intervention consisting of three sessions per week, with at least 48 h between sessions to allow adequate recovery. Each session lasted approximately 45 min and comprised a standardized 10-minute warm-up, the main training phase, and a 10-minute cool-down.

During the main training phase, participants performed one of four protocols (HIIT, HICT, MIIT, or MICT) using predominantly bodyweight-based whole-body movements supplemented by minimal equipment. The exercises were selected to recruit multiple major muscle groups, elicit a whole-body cardiovascular stimulus, allow exercise intensity to be adjusted through movement cadence and effort, and minimize equipment and space requirements. The interval and near-continuous formats were distinguished according to the structure and intended purpose of the interruptions: HIIT and MIIT included systematically prescribed 30-s passive recovery periods after each exercise and 60-s recovery periods between sets, thereby forming an explicit work recovery structure, whereas HICT and MICT involved two consecutive exercises followed by a brief 15–20 s transition, with no planned recovery between sets. Because the protocols required changes in movement, body position, and, in some instances, equipment, these brief transitions were provided to facilitate safe preparation for the subsequent exercise rather than as prescribed recovery intervals; nevertheless, because they may have allowed some physiological recovery, HICT and MICT were operationally regarded as near-continuous rather than completely uninterrupted conditions. Detailed exercise sequences, transition or recovery structures, and set configurations are provided in Supplementary S1.

Furthermore, exercise intensity was prescribed using age-predicted maximal heart rate calculated with the Fox equation (HR_max_ = 220 - age), together with RPE. The target intensity was 77%-95% of age-predicted HR_max_ and an RPE of 14–17 for the high-intensity groups and 64%-76% of age-predicted HR_max_ and an RPE of 12–13 for the moderate-intensity groups [34]. During each training session, heart rate was monitored using a Polar heart-rate sensor (T1-Coded, Finland), while RPE was assessed concurrently as a complementary measure of internal exercise load; when either measure deviated from the prescribed range, instructors provided immediate verbal feedback and instructed participants to adjust their movement pace or effort toward the target intensity.

HRpeak measured during the baseline Bruce graded exercise test was available but was not used prospectively to prescribe training intensity. Therefore, a post hoc verification analysis was conducted among the 38 participants who completed both the baseline and post-intervention assessments. For each participant, the protocol-defined target heart-rate zone was retrospectively reconstructed using the Fox-equation-based intensity ranges described above. The reconstructed lower and upper boundaries were then expressed as percentages of the measured baseline HRpeak. The difference between age-predicted HRmax and measured HRpeak and the number and proportion of participants whose reconstructed upper boundary exceeded measured HRpeak were summarized descriptively.

### Body composition measurement

On the day of measurement, participants underwent body composition assessment in the morning after an overnight fast, with restricted fluid intake for at least 8 h, and following adequate rest. Prior to the assessment, participants were instructed to remove watches, jewelry, and all metallic objects to avoid interference with the results. To standardize dietary and activity conditions, participants were required to maintain their usual dietary habits on the day before testing and were allowed to engage only in routine light physical activity. Height was measured using an OKenjoy TXT-037 intelligent stadiometer (Beijing Aokangda, China). Body composition was assessed using dual-energy X-ray absorptiometry (DXA) (GE Lunar Prodigy, USA), including body weight, fat mass, fat-free mass, visceral adipose tissue (VAT), and body mass index (BMI) [47]. After calibration of the device according to the manufacturer’s instructions, participants’ basic information, including height, age, and sex, was entered into the system [48].

### Cardiorespiratory endurance

Before the test, the Cortex MetaMax II gas analysis system (Cortex, Germany) was calibrated using standard environmental conditions and gases of known concentrations [49]. Participants first performed 5 min warm-up consisting of light jogging at 2.7 km/h with 0% incline, followed by 3 min of dynamic stretching. Subsequently, participants were fitted with an appropriately sized face mask, and an airtightness check was conducted. They then remained seated for 5 min to establish resting metabolic baseline values. The formal test was conducted using the classical Bruce treadmill incremental protocol, with an initial workload of 2.7 km/h at a 10% incline, and both speed and incline were increased simultaneously every 3 min until volitional exhaustion was reached [50]. Throughout the test, respiratory gas exchange data were continuously measured on a breath-by-breath basis using the gas analysis system, and the RPE was recorded at the end of each stage. The criteria for exhaustion included: (1) a respiratory exchange ratio (RER) ≥ 1.10; (2) attainment of at least 90% of age-predicted maximal heart rate; (3) an RPE score ≥ 18; (4) the presence of an oxygen uptake plateau. However, as not all participants exhibited a clear oxygen uptake plateau, the highest 30-second average oxygen uptake value obtained prior to test termination was defined as VO_₂peak_ to objectively assess cardiorespiratory fitness. After the test, participants performed 5 min cool-down at low intensity (2.7 km/h, 0% incline) until physiological parameters returned toward baseline levels.

### Cardiovascular responses

Throughout the experiment, the Bruce incremental protocol was used to induce cardiac stress, and hemodynamic parameters were continuously monitored using a Tango M2 exercise blood pressure monitor (SunTech Medical, USA) [51]. Resting heart rate and resting blood pressure were measured after participants had been seated for 5 min in a quiet, temperature-controlled environment, and the average of two measurements was recorded. Peak parameters, including peak heart rate (HR_peak_) and peak blood pressure, were obtained approximately 60 s before the end of each stage. Blood pressure measurements were conducted using an Oscillometric method synchronized with the electrocardiographic signal to ensure accuracy during high-intensity exercise. In addition, the rate–pressure product (RPP) was calculated to indirectly assess myocardial oxygen consumption and cardiac workload [52]:

$$\:\mathrm{RPP=}\frac{\mathrm{HR}\times\mathrm{SBP}}{\mathrm{1000}}$$

### Statistical analysis

All statistical analyses were performed using SPSS version 27.0 and R version 4.5.1 (effsize package). The normality of continuous variables was assessed using the Shapiro–Wilk test. Normally distributed data are presented as mean ± standard deviation (SD), whereas non-normally distributed data are presented as median and interquartile range (IQR). Baseline differences among groups were assessed using one-way analysis of variance (ANOVA) for normally distributed variables and the Kruskal–Wallis H test for non-normally distributed variables.

Linear mixed-effects models (LMM) with participant-specific random intercepts were used as the primary models for evaluating intervention effects. Each model included fixed effects for group, time, and the group × time interaction. The omnibus F statistics, partial eta squared (η^2^) values, and P values for the group × time interactions reported in Table 2 were derived exclusively from the LMM. Within-group pre-to-post comparisons were based on model-estimated marginal means.For outcomes showing a nominally significant group × time interaction, conditional post-hoc between-group analyses were performed to explore the source of the interaction. Change scores were calculated as Δ = post − pre. For normally distributed change scores, one-way analysis of covariance (ANCOVA) was performed, with change score as the dependent variable, group as the fixed factor, and baseline value as the covariate. For non-normally distributed change scores, the Kruskal–Wallis H test was used. For ANCOVA-based pairwise comparisons, the mean difference (MD) represents the baseline-adjusted between-group difference in change scores. These conditional post-hoc results are presented separately in Fig. 3 and were not used to generate the omnibus statistics reported in Table 2.

Effect sizes were calculated using Hedges’g, with thresholds interpreted as follows: trivial (< 0.2), small (0.2 to 0.5), moderate (0.5 to 0.8), and large (> 0.8). The effect size for interaction effects was expressed as partial eta squared (η^2^), with values of 0.01, 0.06, and 0.14 representing small, moderate, and large effects, respectively [53]. The level of statistical significance was set at α = 0.05. P values and 95% confidence intervals (95% CI) were reported to reflect the precision of the effect estimates. Bonferroni correction was applied to pairwise comparisons derived from the estimated marginal means and to the subsequent post-hoc comparisons. The omnibus fixed-effect tests were not adjusted across outcomes; therefore, the reported omnibus P values are unadjusted, and the corresponding interaction findings were interpreted as exploratory.

## Results

**Fig. 2:**
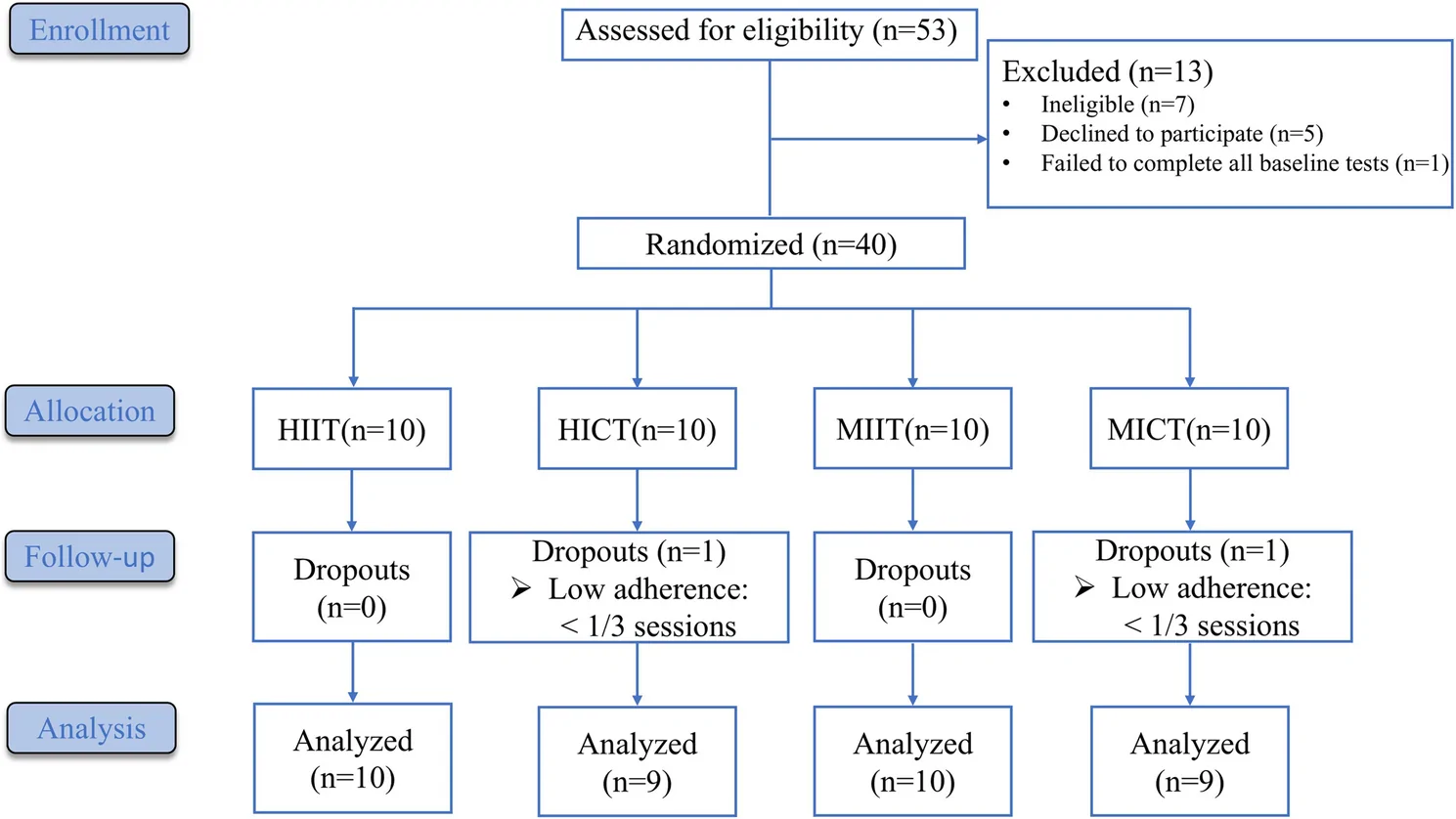
Participant recruitment flowchart

A total of 53 volunteers completed pre-registration during the recruitment phase, and 41 participants were initially screened and entered the baseline testing phase. During the intervention period, one participant withdrew due to personal reasons, and two participants were excluded due to a completion rate of less than one-third of the total training sessions. Ultimately, 38 participants completed the entire experimental protocol. The overall attrition rate was 5%. The study flow is presented in Fig. 2.

As shown in Table 1, no statistically significant baseline differences were observed among the four groups in the measured variables (*P* > 0.05). In a separate post hoc verification analysis, age-predicted HRmax exceeded measured baseline HRpeak, with group-specific mean differences ranging from 9.89 to 14.70 bpm (Electronic Supplementary S3). Compared with reference zones retrospectively recalculated from measured baseline HRpeak using the same percentage boundaries, the Fox-equation-based prescribed zones were shifted upward by 6.33–11.32 bpm at the lower boundary and by 7.52–13.97 bpm at the upper boundary. The Fox-based upper boundary exceeded measured baseline HRpeak in 3 of 9 participants in the HICT group and 7 of 10 participants in the HIIT group, but in none of the participants in the MICT or MIIT groups.

**Table 1:** Comparison of baseline characteristics of participants in different groups

	HICT(*n* = 9)	HIIT(*n* = 10)	MICT(*n* = 9)	MIIT(*n* = 10)	*p*
Years	22.70 ± 2.75	23.10 ± 2.38	24.20 ± 2.10	23.30 ± 2.54	0.58
Height(cm)	178.16 ± 5.60	177.28 ± 7.44	175.33 ± 6.87	171.29 ± 5.73	0.10
Weight(kg)	76.67 ± 7.24	76.54 ± 17.47	77.58 ± 17.91	73.94 ± 11.50	0.86
Fat mass(kg)	20.51 ± 5.25	21.02 ± 5.28	21.61 ± 8.44	19.94 ± 4.08	0.93
Fat-free mass(kg)	56.15 ± 3.61	55.52 ± 12.61	55.96 ± 10.57	54.01 ± 8.35	0.84
BMI(kg/m^2^)	24.37 ± 2.56	24.16 ± 3.88	25.09 ± 5.07	25.10 ± 3.15	0.94
VAT(g)	191.0(139.0–363.3)	247.5(195.3–340.0)	276.5(118.3–817.3)	432.0(196.0–635.3)	0.63
VO_2_Peak(ml/kg/min)	41.71 ± 2.52	42.61 ± 2.59	42.36 ± 2.62	42.27 ± 3.02	0.96
HR_rest_(bpm)	73.44 ± 4.67	74.30 ± 6.36	74.00 ± 5.00	76.20 ± 6.76	0.74
SBP_rest_(mmHg)	120.67 ± 5.15	120.50 ± 7.59	122.78 ± 9.83	123.10 ± 5.41	0.69
DBP_rest_(mmHg)	68.33 ± 7.42	68.70 ± 5.52	73.56 ± 6.37	72.20 ± 7.38 72.20	0.20

Data are presented as mean ± SD or median (P25 - P75)

*BMI* Body Mass Index, *VAT* Visceral Adipose Tissue, *VO*_2Peak_ Peak Oxygen Uptake, *HR*_rest_ Resting Heart Rate, *SBP*_rest_ Resting Systolic Blood Pressure, *DBP*_rest_ Resting Diastolic Blood Pressure

As shown in Table 2, within-group comparisons indicated decreases in BMI in all four groups and decreases in visceral adipose tissue (VAT) in the HICT, HIIT, and MICT groups. No nominal group×time interactions were identified for any body-composition outcome.

**Table 2:** The effect of the intervention on all indicators before and after

Indicators	Time	HICT (*n* = 9)	HIIT (*n* = 10)	MICT (*n* = 9)	MIIT (*n* = 10)	Group×Times		
						F	η^2^	*P*
Weight(kg)	Pre	76.67 ± 7.24	76.54 ± 17.47	77.58 ± 17.91	73.94 ± 11.50	0.051	0.01	0.985
	Post	75.54 ± 7.10	75.52 ± 15.72	76.64 ± 16.71	73.19 ± 10.72			
	∆(95%CI)	-1.13 (-2.57, 0.32)	-1.02 (-2.39, 0.35)	-0.93 (-2.38, 0.51)	-0.75 (-2.12, 0.62)			
Fatmass(kg)	Pre	20.51 ± 5.25	21.02 ± 5.28	21.61 ± 8.44	19.94 ± 4.08	0.116	0.01	0.950
	Post	19.00 ± 4.65	19.77 ± 4.94	20.51 ± 6.28	19.03 ± 2.42			
	∆(95%CI)	-1.51 (-3.01, 0.06)	-1.25 (-2.80, 0.30)	-1.10 (-2.73, 0.53)	-0.90 (-2.45, 0.65)			
Fat free mass(kg)	Pre	56.15 ± 3.61	55.52 ± 12.61	55.96 ± 10.57	54.01 ± 8.35	0.067	0.01	0.977
	Post	56.54 ± 4.12	55.75 ± 11.82	56.13 ± 11.64	54.16 ± 9.20			
	∆(95%CI)	0.39 (-0.47, 0.79)	0.23 (-0.58, 1.05)	0.17 (-0.69, 1.03)	0.15 (-0.66, 0.97)			
BMI(kg/m^2^)	Pre	24.37 ± 2.56	24.16 ± 3.88	25.09 ± 5.07	25.10 ± 3.15	0.158	0.04	0.924
	Post	23.87 ± 2.47	23.73 ± 3.53	24.70 ± 4.61	24.75 ± 2.74			
	∆(95%CI)	-0.49(-0.82, -0.17)**	-0.43(-0.74, -0.11)**	-0.39(-0.72, -0.07)*	-0.34(-0.66, -0.03)*			
VAT(g)	Pre	191(139–363.3)	247.5(195.3–340)	276.5(118.3–817.3)	432(196–635.3)	1.370	0.12	0.273
	Post	124(93.5–260)	205(158.3–290.5)	284(88–820.5)	408(171.3–597.8)			
	∆(95%CI)	-47.5(-64,-28.5)*	-44.5(-63.5, -34.5)*	-32.5(-68, -7.5)*	-27(-44, 0)			
VO_2_Peak(ml/kg/min)	Pre	41.71 ± 2.52	42.61 ± 2.59	42.36 ± 2.62	42.27 ± 3.02	3.098	0.21	0.04
	Post	43.780 ± 2.17	44.95 ± 2.19	44.20 ± 2.65	43.55 ± 2.63			
	∆(95%CI)	2.07 (1.52, 2.61)**	2.34 (1.82, 2.86)**	1.84 (1.30, 2.39)**	1.28 (0.76, 1.80)**			
Bruce test duration(min)	Pre	10.04 ± 0.92	10.26 ± 1.04	10.18 ± 1.05	10.88 ± 1.27	3.980	0.26	0.016
	Post	11.28 ± 0.58	11.36 ± 1.08	11.03 ± 0.90	11.40 ± 1.09			
	∆(95%CI)	1.24(0.91, 1.58)**	1.10(0.79, 1.42)**	0.85(0.51, 1.19)**	0.52(0.20, 0.84)**			
HR_rest_(bpm)	Pre	73.44 ± 4.67	74.30 ± 6.36	74.00 ± 5.00	76.20 ± 6.73	2.657	0.21	0.064
	Post	69.44 ± 3.40	70.10 ± 3.96	72.33 ± 3.43	75.00 ± 4.83			
	∆(95%CI)	-4.00 (-6.00, -1.99)**	-4.20 (-6.10, -2.30)**	-1.67 (-3.06, 0.34)	-1.20 (-3.10, 0.70)			
SBP_rest_(mmHg)	Pre	120.67 ± 5.15	120.50 ± 7.59	122.78 ± 9.83	123.10 ± 5.41	8.883	0.44	< 0.001
	Post	115.11 ± 4.81	115.10 ± 6.30	121.33 ± 7.75	122.00 ± 5.19			
	∆(95%CI)	-5.56(-7.26, -3.85)**	-5.40(-7.02, -3.78)**	-1.44(-3.15, 0.26)	-1.10(-2.72, 0.52)			
DBP_rest_(mmHg)	Pre	68.33 ± 7.42	68.70 ± 5.52	73.56 ± 6.37	72.20 ± 7.38	0.607	0.05	0.615
	Post	66.78 ± 6.38	67.00 ± 4.88	72.56 ± 5.86	71.50 ± 7.03			
	∆(95%CI)	-1.56(-2.83, -0.28)*	-1.70(-2.91, -0.49)**	-1.00(-2.28, 0.28)	-0.70(-1.91, 0.51)			
RPP_rest_(mmHg·bpm)	Pre	8.86 ± 0.71	8.97 ± 1.08	9.10 ± 1.08	9.38 ± 0.88	7.402	0.40	< 0.001
	Post	7.99 ± 0.52	8.08 ± 0.79	8.78 ± 0.81	9.15 ± 0.72			
	∆(95%CI)	-0.87(-1.14, -0.60)**	-0.89(-1.15, -0.63)**	-0.31(-0.59, -0.04)*	-0.23(-0.48, 0.33)			
HR_peak_(bpm)	Pre	186.11 ± 13.71	182.20 ± 12.87	185.78 ± 9.78	182.50 ± 12.41	2.474	0.18	0.078
	Post	183.56 ± 9.30	179.70 ± 10.44	186.67 ± 8.87	183.80 ± 10.29			
	∆(95%CI)	-2.56 (-5.35, -0.24)	-2.50(-5.15, 0.15)	0.89(-1.90, 3.68)	1.30(-1.35, 3.95)			
SBP_peak_(mmHg)	Pre	186.44 ± 7.67	190.10 ± 7.75	179.67 ± 11.93	185.40 ± 9.08	6.687	0.36	0.001
	Post	178.44 ± 6.23	182.90 ± 7.05	178.89 ± 7.75	183.80 ± 7.21			
	∆(95%CI)	-8.00(-10.99, -5.01)**	-7.20(-10.04, -4.36)**	-0.78(-3.77, 2.22)	-1.60(-4.44, 1.24)			
DBP_peak_(mmHg)	Pre	76.44 ± 7.44	75.10 ± 8.28	77.44 ± 6.50	79.20 ± 8.51	1.547	0.12	0.220
	Post	74.22 ± 7.23	72.10 ± 7.80	76.00 ± 5.27	77.90 ± 7.58			
	∆(95%CI)	-2.22(-3.15, -1.29)**	-2.40(-3.28, -1.52)**	-1.44(-2.38, -0.51)**	-1.30(-2.18, -0.42)**			
RPP_peak_(mmHg·bpm)	Pre	34.74 ± 3.40	34.65 ± 3.09	33.41 ± 3.18	33.83 ± 2.67	8.275	0.42	< 0.001
	Post	32.78 ± 2.44	32.88 ± 2.48	33.42 ± 2.53	33.77 ± 2.14			
	∆(95%CI)	-1.96(-2.73, -1.19)**	-1.78(-2.51, -1.04)**	0.01(-0.76, 0.78)	-0.05(-0.79, 0.68)			

Data are presented as mean ± SD or median (P25–P75). The F statistics, η^2^ values, and P values represent the omnibus group×time interaction effects derived from the LMMs. Omnibus P values were not adjusted across outcomes and should be interpreted as exploratory. Changes from pre- to post-intervention were estimated using model-derived marginal means. *P < 0.05 and **P < 0.01 for Bonferroni-adjusted within-group pre-to-post comparisons.; bruce test duration: the total time sustained during the graded exercise test; RPP_rest_: Resting Rate-Pressure Product; HR_peak_: Peak Heart Rate; SBP_Peak_: Peak Systolic Blood Pressure; DBP_Peak_: Peak Diastolic Blood Pressure; RPP_Peak_: Peak Rate-Pressure Product

**Fig. 3:**
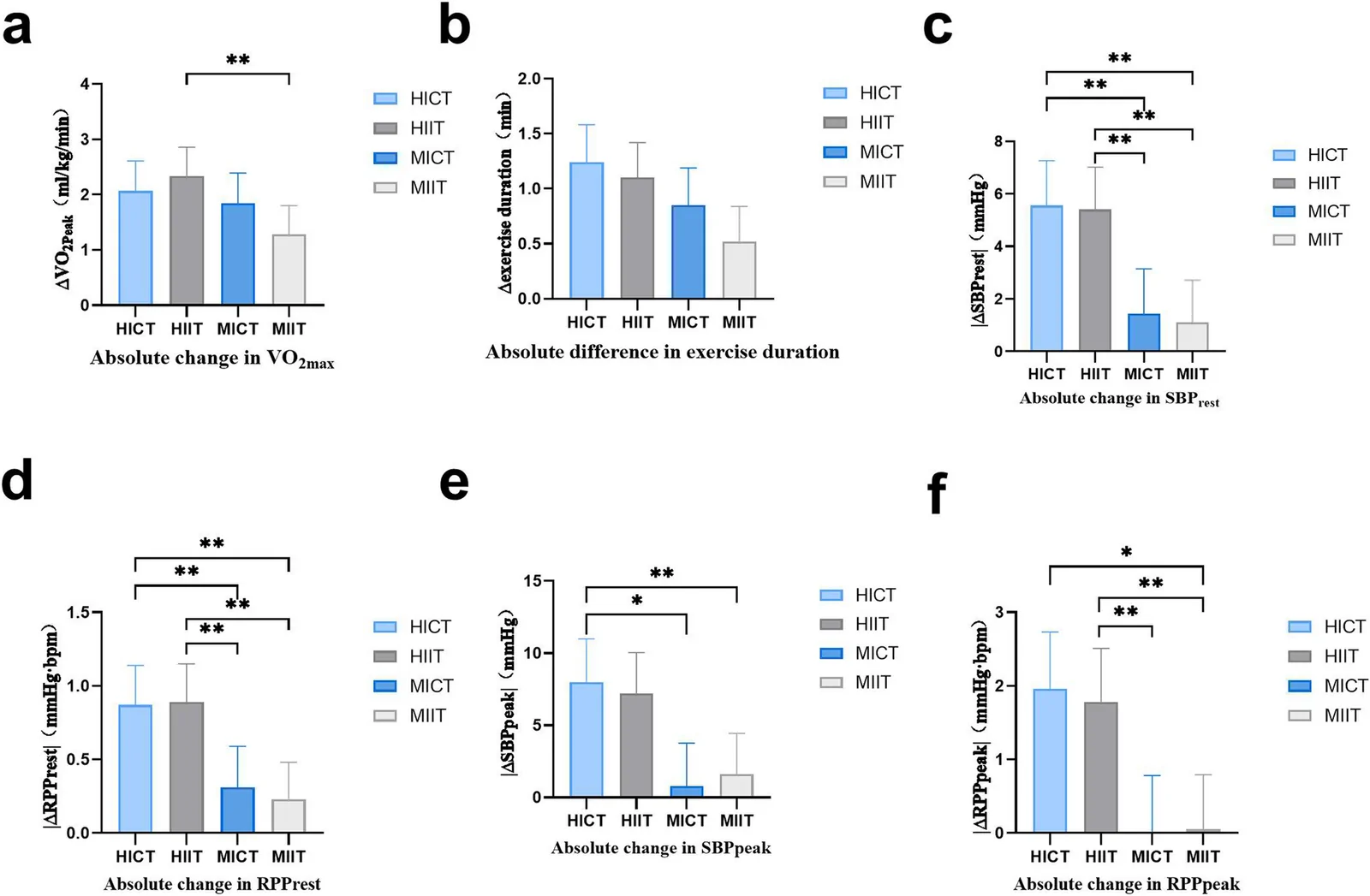
Absolute changes in selected cardiorespiratory and hemodynamic outcomes following the 8-week exercise interventions. **a** Absolute change in VO₂peak; **b** absolute difference in Bruce graded exercise test duration; **c** absolute change in resting systolic blood pressure (SBPrest); **d** absolute change in resting rate-pressure product (RPPrest); **e** absolute change in peak systolic blood pressure (SBPpeak); and **f** absolute change in peak rate-pressure product (RPPpeak). Data are presented as mean ± SD. HICT, high-intensity continuous training; HIIT, high-intensity interval training; MICT, moderate-intensity continuous training; MIIT, moderate-intensity interval training. *P < 0.05; **P < 0.01

Table 2 reports the group×time interaction results for each outcome. The principal between-group comparisons for outcomes showing nominal interactions are illustrated in Fig. 3. Complete LMM results for all outcomes, including the main effects of group and time and the group×time interaction, together with all pairwise comparisons reporting MD, 95% CI, Hedges’g, and Bonferroni-adjusted P values, are provided in Electronic Supplementary S2.

For cardiorespiratory fitness, within-group comparisons indicated increases in VO_2_peak and Bruce test duration in all four groups. Exploratory LMM analyses identified nominal group × time interactions for VO_2_peak (F = 3.098, η² = 0.21, *P* = 0.040) and Bruce test duration (F = 3.980, η² = 0.26, *P* = 0.016). In the conditional post-hoc analyses, the increase in VO_2_peak was greater in HIIT than in MIIT after Bonferroni correction (MD = 1.11 mL·kg⁻¹·min⁻¹; 95% CI [0.20, 2.01], g = 1.54; *P* < 0.01). However, for Bruce test duration none of the subsequent Bonferroni-adjusted pairwise between-group comparisons reached statistical significance. The largest estimated difference was observed between HICT and MIIT (MD = 0.54 min, 95% CI [-0.05, 1.13], g = 1.22), although the confidence interval included zero.

Cardiovascular outcomes were examined separately at rest and during peak exercise. Exploratory LMM analyses identified nominal group×time interactions for resting systolic blood pressure (SBP_rest_; F = 8.883, η² = 0.44, *P* < 0.001), resting rate pressure product (RPP_rest_; F = 7.402, η² = 0.40, *P* < 0.001), peak systolic blood pressure (SBP_peak_; F = 6.687, η² = 0.36, *P* = 0.001), and peak rate pressure product (RPP_peak_; F = 8.275, η² = 0.42, *P* < 0.001). post-hoc analyses (Fig. 3)showed greater reductions in SBP_rest_ and RPP_rest_ in the HICT and HIIT groups than in the MICT and MIIT groups. The largest differences were observed between HICT and MIIT for SBP_rest_ (MD = -4.95 mmHg, 95% CI [-7.65, -2.25], g = -1.70) and RPP_rest_ (MD = -0.80 mmHg·bpm, 95% CI [-1.18, -0.42], g = -1.61). The reduction in SBP_peak_ was greater in HICT than in MICT and MIIT, with the largest difference observed between HICT and MIIT (MD = -6.07 mmHg, 95% CI [-10.38, -1.76], g = -1.94). Reductions in RPP_peak_ were greater in HICT and HIIT than in the moderate-intensity groups, with the largest difference observed between HIIT and MIIT (MD = -0.77 mmHg·bpm, 95% CI [-1.26, -0.28], g = -1.79). These interaction findings were considered exploratory because the omnibus tests were not adjusted across outcomes.

## Discussion

Overall, several outcomes improved over time across the four training conditions. No significant group-by-time interactions were observed for body composition outcomes. In contrast, significant interactions were detected for selected cardiorespiratory fitness and cardiovascular outcomes, with some between-group comparisons showing greater improvements in the high-intensity groups. These findings suggest a potential advantage of higher-intensity training for specific cardiorespiratory and cardiovascular outcomes. However, training adaptations may reflect the combined influence of exercise intensity, training format, energy expenditure, and other factors. Therefore, the findings for body composition, cardiorespiratory fitness, and cardiovascular responses are discussed separately below in relation to the outcome-specific results and relevant evidence.

### Body composition

Regarding body composition, significant changes over time were observed for BMI and VAT, whereas no significant group-by-time interactions were detected for body weight, fat mass, fat-free mass, BMI, or VAT. Thus, although BMI and VAT changed during the study period, the magnitude of these changes could not be statistically differentiated among the four training conditions. The small differences in the absolute change estimates, such as the reduction in BMI ranging from 0.34 to 0.49 kg/m^2^ across groups, should not be interpreted as evidence that one training intensity or format was superior, particularly given the overlapping confidence intervals and the small number of participants in each group. Moreover, statistical significance of a change over time does not necessarily indicate clinical meaningfulness. Because prespecified thresholds for clinically meaningful changes in these body-composition outcomes were not evaluated, the clinical relevance of the observed changes remains uncertain. In addition, without a non-exercise control group, the temporal changes cannot be attributed exclusively to the training interventions, because concurrent influences such as habitual behavior changes, seasonal variation, or regression to the mean cannot be excluded.

The absence of differential effects on body composition is broadly consistent with meta-analyses reporting no clear advantage of HIIT over MICT for reducing BMI or fat mass [14, 26, 54]. However, other studies have reported greater reductions in adiposity following higher-intensity training [55–57]. Rather than establishing either position as definitive, the mixed evidence suggests that body-composition responses may depend on factors such as participants’ baseline weight status and sex, intervention duration, exercise modality, dietary behavior, and the method used to match training exposure. The present findings therefore contribute to this literature by showing that, among sedentary young men completing an 8 weeks bodyweight-based and time-matched intervention, the relative effects of exercise intensity and interval versus continuous format on body composition were not clearly distinguishable. Meanwhile, An important consideration is that the intervention was matched for exercise time rather than for mechanical work or energy expenditure. Although each movement was performed for a total of 120 s, the high- and moderate-intensity protocols differed in work-bout structure and physiological load. Higher-intensity exercise may produce greater acute energy expenditure, excess post-exercise oxygen consumption, and neuroendocrine responses than moderate-intensity exercise performed for the same duration [58–61]. These differences could theoretically influence body-composition adaptation; however, energy expenditure, post-exercise oxygen consumption, and related metabolic responses were not measured in this study. They should therefore be considered possible sources of variation in the intervention dose rather than mechanisms demonstrated by the present findings. Conversely, the absence of significant interactions indicates that any differences in physiological load did not translate into statistically detectable differences in body composition over the 8 weeks intervention. However, Uncontrolled dietary intake provides another plausible explanation for the limited separation among groups. Body weight and adiposity are strongly influenced by energy balance, and participants were instructed to maintain their habitual diet rather than being provided with a controlled diet or undergoing systematic dietary assessment. Changes in appetite or compensatory energy intake after exercise may therefore have attenuated, obscured, or increased the variability of the body-composition response [62, 63]. In addition, the 8 weeks intervention and the sample of only 9–10 participants per group limited the ability to detect small between-group effects. The non-significant interactions should consequently be interpreted as an absence of evidence for differential effects under the present conditions, rather than evidence that training intensity and format have equivalent effects in all settings.

Taken together, the present study does not provide statistical evidence that higher exercise intensity or either interval or continuous training produces superior body-composition outcomes. It instead indicates that the relative effects of the four protocols could not be clearly differentiated within this small, 8 weeks, time-matched trial.

### Cardiorespiratory fitness

The cardiorespiratory fitness responses differed according to the outcome assessed. Although VO_2_peak and Bruce test duration increased over time across all four training conditions, the between-group findings were less uniform. HIIT produced a greater improvement in VO_2_peak than MIIT, suggesting that combining high intensity with an interval structure may provide an additional stimulus for maximal aerobic capacity. However, this pattern was not observed consistently across the other training comparisons. Bruce test duration also showed an overall differential response among the four conditions, but no individual pairwise difference was identified after adjustment for multiple comparisons. Overall, different training protocols may influence different aspects of cardiorespiratory fitness. For example, HIIT may be more beneficial for improving VO_2_peak, whereas no training protocol showed a clear advantage for Bruce test duration.

Previous evidence remains inconsistent regarding whether HIIT produces greater improvements in VO_2_max or VO_2_peak than moderate-intensity continuous training. Some systematic reviews and meta-analyses have reported a greater average improvement following HIIT, although the additional benefit is generally modest [25, 27, 64, 65], whereas other studies have found broadly comparable cardiorespiratory adaptations following interval and continuous training [66]. The present study showed a similarly mixed pattern: HIIT improved VO_2_peak more than MIIT, but this advantage was not observed in the other between-group comparisons. Thus, HIIT may be particularly beneficial for VO_2_peak under certain conditions, but the findings do not indicate that interval or high-intensity training is more effective for every aspect of cardiorespiratory fitness. Notably, HICT also showed appreciable cardiorespiratory adaptation, suggesting that the effects of a high-intensity stimulus may not depend entirely on a conventional interval structure.Despite using a near-continuous format rather than the conventional work–recovery structure of HIIT, HICT was accompanied by increases in both VO_2_peak and Bruce test duration, and its change in VO_2_peak did not differ significantly from either HIIT or MICT. Although this finding does not establish equivalence among these protocols, it suggests that near-continuous high-intensity bodyweight training may also provide a sufficient physiological stimulus for cardiorespiratory adaptation. In other words, adaptations commonly observed following HIIT may arise not only from its interval structure but also from the accumulation of a high-intensity stimulus during training. The HICT findings therefore extend the conventional HIIT and MICT comparison and provide a rationale for evaluating near-continuous high-intensity training as a distinct exercise strategy. Furthermore, The outcome-specific responses may reflect differences in how the protocols accumulated cardiovascular and metabolic stress. Repeated high-intensity bouts during HIIT may allow participants to repeatedly reach a high oxygen demand while using recovery periods to sustain subsequent work, a pattern associated in previous literature with central and peripheral adaptations relevant to VO_2_peak [30–33, 67, 68]. In contrast, the near-continuous structure of HICT may maintain an elevated physiological demand for a more sustained period, which could favor tolerance of prolonged exercise and help explain the observed increase in Bruce test duration [69–71]. These explanations should be regarded as literature-based interpretations rather than direct findings of the present study. In addition, the increases observed across the four groups indicate that whole-body, predominantly bodyweight-based exercise can provide a meaningful cardiorespiratory stimulus without reliance on treadmills or cycle ergometers. This is consistent with previous evidence supporting the feasibility and cardiorespiratory benefits of whole-body bodyweight training [66, 72].

In a word, the cardiorespiratory findings are outcome- and comparison-specific. HIIT produced a greater improvement in VO_2_peak than MIIT, but no consistent superiority of high-intensity over moderate-intensity training or of interval over near-continuous training was demonstrated across all comparisons. The HICT response is nevertheless an important finding, as it suggests that a near-continuous high-intensity bodyweight protocol may also elicit cardiorespiratory adaptation. These results favor a more nuanced interpretation in which exercise intensity and training format may contribute differently depending on the selected cardiorespiratory outcome.

### Cardiovascular responses

This study systematically compared changes in HR, BP, and RPP at rest and during peak exercise across different training intensities and formats. Overall, the most distinct differences among the four training conditions were observed for systolic blood pressure and RPP, with the high-intensity groups generally showing greater reductions than the moderate-intensity groups at both stages. In contrast, the changes in HR and diastolic blood pressure were less clearly differentiated among the training conditions. These findings are broadly consistent with previous studies [32, 73–78] and provide population-specific evidence that training intensity may influence selected cardiovascular responses in sedentary male college students. The favorable responses observed following both HIIT and HICT further suggest that such adaptations are not restricted to a single training format.

Notably, relatively large between-group effect sizes were observed for selected SBP and RPP outcomes. However, when considered in absolute terms, the between-group differences in SBP observed in the present study (4.95–6.07 mmHg) were within a physiologically plausible range compared with previous exercise interventions. For example, Arboleda-Serna et al. reported an 8-mmHg difference in post-intervention resting SBP between MICT and HIIT after 8 weeks, although the difference favored MICT rather than HIIT [79]. Similarly, Shandu et al. found that 8 weeks of HIIT and continuous aerobic training reduced resting RPP by 10.1% and 10.8%, respectively, in college-aged male smokers, despite no significant changes in resting SBP [80]. Thus, the large standardized effects observed in the present study do not necessarily indicate implausibly large changes in absolute cardiovascular outcomes. Nevertheless, because the present estimates were derived from only 9–10 participants per group, they may be unstable and potentially inflated, particularly in the presence of relatively low within-group variability, and should therefore be interpreted cautiously.

After the 8 weeks intervention, the greater reductions in resting systolic blood pressure and RPP in the high-intensity groups suggest more favorable adaptations in resting circulatory regulation and myocardial workload. Previous studies have associated higher-intensity exercise with enhanced parasympathetic activity and reduced sympathetic drive [62, 73, 74]. Such autonomic adaptations may contribute to more efficient cardiovascular regulation, although the present findings showed no clear between-group differentiation in resting HR. The reduction in resting systolic blood pressure may also be related to adaptations in endothelial function and vascular compliance. The repeated elevations in blood flow and shear stress induced by high-intensity exercise may enhance endothelial responsiveness and promote nitric oxide-mediated vasodilation, thereby reducing peripheral resistance [67, 78, 81]. In addition, the reduction in resting RPP suggests a lower estimated myocardial oxygen demand. Because RPP reflects the combined influence of HR and systolic blood pressure, its more pronounced reduction in the high-intensity groups may indicate improved cardiac efficiency under resting conditions, which is consistent with previous observations concerning cardiovascular responses to interval and continuous exercise [68].

During peak exercise, the greater reductions in systolic blood pressure and RPP following high-intensity training suggest that the cardiovascular system may have adapted to manage a demanding exercise load with a lower pressure response and myocardial workload. A lower peak systolic blood pressure may reflect improved regulation of peripheral vascular resistance and a more economical cardiovascular response during maximal exertion. This interpretation is consistent with evidence linking exercise-induced vascular adaptation with improved vasodilation and blood-pressure regulation [74, 77, 78, 82]. The accompanying reduction in peak RPP further suggests a lower estimated cardiac metabolic cost during maximal exercise. Previous research has shown that, although high-intensity exercise can acutely increase cardiovascular demand, repeated training may lead to more efficient chronic cardiovascular responses [68]. In the present study, both HICT and HIIT showed favorable changes in selected cardiovascular outcomes, suggesting that high-intensity training may promote cardiovascular adaptation when delivered in either a near-continuous or interval format.

### Limitations and suggestions

The four-arm design enabled the responses to different combinations of exercise intensity and training format to be compared within the same study, while exercise time was standardized across groups. The findings suggest that higher-intensity training may have an advantage for selected outcomes: HIIT produced a greater improvement in VO_2_peak than MIIT, and the high-intensity protocols showed greater reductions in selected systolic blood pressure and RPP outcomes. However, this pattern was not consistent across all measures. No differential effects were detected for body composition, no adjusted pairwise differences were found for Bruce test duration, and interval training was not consistently superior to near-continuous training. Exercise prescription should therefore be guided by the specific outcome of interest rather than by assuming that one training protocol is universally superior. When these findings are considered in practice, time requirements, individual tolerance, preference, safety, and adherence are also important. Previous evidence suggests that low-volume HIIT can improve cardiorespiratory fitness and blood pressure with less training time than traditional MICT, which may make it a useful option for young adults with limited time [83–85]. Nevertheless, the present study did not directly compare the time efficiency, enjoyment, or long-term adherence associated with the four protocols. HICT also produced favorable changes in selected cardiorespiratory and cardiovascular outcomes, indicating that potentially beneficial high-intensity stimuli can be delivered through either an interval or a near-continuous format. Accordingly, HIIT, HICT, MIIT, and MICT should be viewed as alternative strategies whose suitability may vary according to the intended outcome and the characteristics of the individual, rather than as a fixed hierarchy in which one modality should always be prioritized.

Several limitations should be acknowledged. First, only 38 participants completed the intervention, leaving 9–10 participants in each group. This limited the precision of the effect estimates and the ability to detect smaller between-group differences, particularly after adjustment for multiple comparisons. Non-significant comparisons therefore do not establish that the protocols were equivalent. In addition, the absence of a non-exercise control group means that changes over time cannot be attributed exclusively to the interventions. Although the between-group comparisons remain useful for evaluating relative responses, concurrent behavioral changes, seasonal influences, or regression to the mean cannot be excluded. Dietary intake was also not systematically assessed or controlled, which may have increased variability in the body-composition responses and obscured differences among groups. Second, exercise intensity was prescribed using age-predicted HRmax derived from the Fox equation rather than HRpeak measured during the baseline Bruce graded exercise test. The post hoc verification analysis showed that age-predicted HRmax exceeded measured baseline HRpeak by group-specific averages of 9.9–14.7 bpm. Consequently, the Fox-equation-based prescribed zones were shifted upward relative to reference zones retrospectively recalculated from measured baseline HRpeak, particularly in the high-intensity groups, in which the prescribed upper boundary exceeded measured baseline HRpeak in 10 of 19 participants. These findings indicate that the use of age-predicted HRmax may have introduced individual error and reduced the precision of intensity classification. However, the reconstructed zones represent prescribed target ranges rather than actual session-level exercise intensity, and HRpeak measured during a graded exercise test may not necessarily represent true maximal heart rate. Future studies should prescribe exercise intensity using individualized physiological measures, such as measured HRpeak, heart-rate reserve, or ventilatory thresholds, and retain session-level intensity data to verify intervention fidelity. Third, the physiological explanations proposed in the Discussion, including autonomic regulation, endothelial function, cardiac efficiency, and mitochondrial adaptation, were derived from previous literature because these mechanisms were not directly measured. Moreover, all participants were sedentary young men, limiting generalizability to women, other age groups, clinical populations, and individuals with different activity or body-weight profiles. The 8 weeks intervention and absence of post-intervention follow-up also prevent conclusions about the persistence of the observed changes. Future studies should use larger and more diverse samples, include a non-exercise or usual-activity control group, prescribe intensity using individualized physiological measures, directly quantify mechanical work and energy expenditure, assess dietary intake and intervention fidelity, and include longer follow-up. Measures of enjoyment, safety, preference, and adherence would also help determine how these protocols can be applied in university and community settings. Finally, the small group sizes (*n* = 9–10) may have resulted in unstable and potentially inflated standardized effect-size estimates. Therefore, the very large Hedges’g values observed for selected SBP and RPP comparisons should be interpreted as preliminary and require confirmation in adequately powered studies with larger samples.

Taken together, the present study provides preliminary evidence that the relative effects of exercise intensity and training format differ according to the outcome assessed. Higher-intensity training may be useful when improvements in VO_2_peak and selected systolic blood pressure and rate-pressure product responses are the primary targets, whereas the choice between interval and near-continuous formats may remain flexible. These implications should be confirmed before firm recommendations are made regarding which protocol should be prioritized for sedentary young adults.

## Conclusion

This study provides preliminary evidence that the effects of the four training protocols in sedentary young men were outcome-specific. HIIT produced a greater improvement in VO_2_peak than MIIT, while the high-intensity protocols showed greater reductions in selected resting and peak-exercise SBP and RPP outcomes. However, no adjusted between-group differences were detected for Bruce test duration, no differential effects were observed for body composition, and interval training was not consistently superior to near-continuous training. These findings suggest that HIIT and HICT may be considered when targeting VO_2_peak and selected cardiovascular responses, whereas training format may be selected flexibly according to the intended outcome, individual tolerance, and preference.

## Supplementary Information


Supplementary Material 1.


## Data Availability

Data availability statementThe data that support the findings of this study are available from the corresponding author upon reasonable request.

## Abbreviations

HIIT: High-Intensity Interval Training
HICT: High-Intensity Continuous Training
MIIT: Moderate-Intensity Interval Training
MICT: Moderate-Intensity Continuous Training
HRmax: Maximum Heart Rate
RPE: Rating of Perceived Exertion
VO_2_peak: Peak Oxygen Uptake
RPP: Rate Pressure Product
VAT: Visceral Adipose Tissue
